# Ru/C‐Catalyzed Hydrogenation of Aqueous Glycolic Acid from Microalgae – Influence of pH and Biologically Relevant Additives

**DOI:** 10.1002/open.202200050

**Published:** 2022-07-13

**Authors:** Florian M. Harth, Joran Celis, Anja Taubert, Sonja Rössler, Heiko Wagner, Michael Goepel, Christian Wilhelm, Roger Gläser

**Affiliations:** ^1^ Institute of Chemical Technology Universität Leipzig Linnéstr. 3 04103 Leipzig Germany; ^2^ Department of Algal Biotechnology Universität Leipzig Permoserstr. 15 04318 Leipzig Germany

**Keywords:** algal photobiocatalysis, catalyst deactivation, heterogeneous catalysis, hydrogenation, renewable ethylene glycol

## Abstract

Ethylene glycol (EG) is obtained by a novel, two‐step approach combining a biotechnological and a heterogeneously catalyzed step. First, microalgae are cultivated to photobiocatalytically yield glycolic acid (GA) by means of photosynthesis from CO_2_ and water. GA is continuously excreted into the surrounding medium. In the second step, the GA‐containing algal medium is used as feedstock for catalytic reduction with H_2_ to EG over a Ru/C catalyst. The present study focuses on the conversion of an authentic algae‐derived GA solution. After identification of the key characteristics of the algal medium (compared to pure aqueous GA), the influence of pH, numerous salt additives, pH buffers and other relevant organic molecules on the catalytic GA reduction was investigated. Nitrogen‐ and sulfur‐containing organic molecules can strongly inhibit the reaction. Moreover, pH adjustment by acidification is required, for which H_2_SO_4_ is found most suitable. In combination with a modification of the biotechnological process to mitigate the use of inhibitory compounds, and after acidifying the algal medium, over Ru/C a EG yield of up to 21 % even at non‐optimized reaction conditions was achieved.

## Graphical abstract

**Acidified algae‐derived glycolic acid** can be directly converted into ethylene glycol by heterogeneously catalyzed hydrogenation over Ru/C. Yet, catalytic activity is strongly decreased compared to pure glycolic acid. Model experiments show the inhibitory effect of inorganic salts, pH buffers and other organic molecules present in the algal medium on the catalytic reaction.

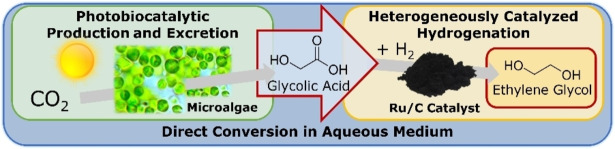

## Introduction

As part of the concerted effort to find sustainable and biomass‐based routes to industrially demanded chemicals, several strategies have been developed in recent years to explore such renewable pathways for the production of glycolic acid (GA). Currently, the α‐hydroxycarboxylic acid GA, which is an important precursor for biodegradable polyester production,^[1]^ is still produced either by carbonylation of formaldehyde^[2]^ or by hydrolysis of monochloroacetic acid.^[3]^ “Conventional” biomass‐based processes rely on the growth, harvesting and subsequent conversion of plant biomass. The conversion of biomass into GA can either be based on (homo‐ or heterogeneously) catalyzed processes (reviewed in Refs. [4,5]; only few studies report starting directly from raw biomass or cellulose^[6]^) or on biotechnological processes (reviewed in Refs. [7,8]). Fermentation with *Escherichia coli* bacteria is most advanced and can yield aqueous product solutions with GA concentrations >50 g L^−1^ (≈700 mmol L^−1^).[^7^, ^9^]

In this study, GA was obtained using the approach of Wilhelm et al.,[^10^, ^11^, ^12^, ^13^] in which algal cells (*Chlamydomonas reinhardtii*) are not used as substrate feedstock, but as direct producers of GA. The algal cells function as “photobioreactors” by exploiting the dual function of the Rubisco enzyme. The absorbed light energy from the photosynthetic electron transport chain is used for glycolate formation through the process of photorespiration. Importantly, the process of photorespiration is initiated by a high cultivation temperature (30–35 °C) and a high O_2_/CO_2_ ratio of aeration.^[11]^ The advantage of this GA‐producing system is that the cells excrete GA directly into the aqueous medium. Therefore, the GA‐containing medium, but not the algal cells themselves are harvested. Consequently, the amount of cell biomass is kept constant and CO_2_ is selectively funneled into the production of GA. So far, reported accumulated GA concentrations did not exceed 3 g L^−1^ or 40 mmol L^−1^,^[11]^ but further improvements can be expected. The concept has considerable advantages. The conversion of CO_2_ into GA is very efficient (up to 82 % of assimilated carbon is directed to glycolate synthesis), separation of GA‐containing aqueous medium and algal cells is rather easy to achieve (as no extraction from the algal cells is needed) and the approach inherently offers the potential of continuous GA production. Furthermore, the amounts of biological impurities are expected to be low compared to fermentation‐based approaches.^[11]^

**Figure 1:**
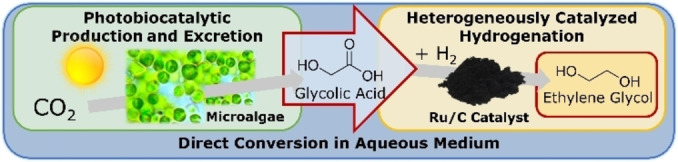
Combination of algae‐based photobiocatalytic formation of glycolic acid from CO_2_ and sunlight with subsequent heterogeneously catalyzed hydrogenation to obtain industrially demanded ethylene glycol.

While biomass‐based GA is becoming increasingly available, separation and purification poses an economical and technological challenge. To circumvent costly multi‐step separation and purification of GA,[^14^, ^15^] its direct conversion into ethylene glycol (EG) is a viable alternative, following a suggestion by Huo and Shanks for similar carboxylic compounds.^[15]^ EG is a highly demanded bulk chemical with several industrial applications, for example, as a polyester precursor, for which biomass‐based pathways are of utmost research interest.[^4^, ^16^] The direct aqueous‐phase conversion of GA into EG is possible with H_2_ as a reducing agent over supported metal catalysts.[^17^, ^18^] Therefore, we propose renewable EG production from CO_2_ in a two‐step process by combining the photobiocatalytic formation of GA and the heterogeneously catalyzed hydrogenation of GA (illustrated in Figure 1).

In a recent study,^[18]^ we systematically investigated supported metal catalysts for the hydrogenation of diluted aqueous GA model solutions (GA concentration of 70 mmol L^−1^, i. e., in the range of state‐of‐the‐art algal media). Ru is an especially suited catalyst among other metals (Pt, Pd, Re) and the support material has a considerable influence on both catalytic activity and selectivity. Diluted GA solutions can be almost quantitatively converted into EG (up to 90 % yield) applying a suitable catalyst (Ru/TiO_2_ or commercial Ru/C) at mild reaction conditions (105 °C, 60 bar H_2_).^[18]^ These results serve as the starting point of the present investigation.

Understanding the challenges from changing the feedstock from pure aqueous GA solutions to real, biotechnologically produced algal media is the key topic addressed here. In general, the combination of biotechnological and subsequent heterogeneously catalyzed processes can present new challenges. Biotechnology‐derived product solutions, for example, fermentation broths or, to a lesser extent, the GA‐containing algal media, contain various amounts of additives and impurities, which can have various detrimental effects on subsequent processing steps.^[15]^

Acids or bases can lead to protonation or deprotonation of reactants. For instance, lactic acid‐containing fermentation broths^[19]^ (around neutral pH) or crude calcium lactate^[20]^ need to be acidified to enable conversion to propanediol over Ru catalysts, a reaction that is analogous to GA hydrogenation. It was suggested that lactic acid is only reactive in the protonated acid form^[20]^ or as “free” lactate (as opposed to calcium lactate).^[19]^ Furthermore, pH changes can also promote undesired side reactions^[21]^ and challenging pH conditions can result in deactivation of solid catalysts.[^22^, ^23^]

The corresponding inorganic ions of typical salts such as K_2_SO_4_ or Na_2_HPO_4_ lower the solubility of gases like H_2_ resulting in decreased hydrogenation activity[^20^, ^24^] but may also lead to poisoning of catalytically active sites. In particular, sulfur‐containing ions were found to interact with active metal centers of a supported Ru catalyst,^[23]^ while Na^+^ and K^+^ were suggested to poison Brønsted acid sites of a bifunctional Ir−ReO_x_/SiO_2_ catalyst.^[25]^ Nutrient solutions additionally contain transition metal ions like Mn Fe, Co or Cu, which could interfere with heterogeneously catalyzed processes, even at only low concentrations of several μmol.^[11]^

Among organic compounds, alanine and other amino acids without sulfur atoms were found to reversibly bind to catalytically active metal sites, that is, the catalytic activity can be recovered^[24]^ and their influence on the desired reaction is comparatively weak.^[23]^ On the other hand, amino acids or other molecules containing thiol or similar sulfur‐containing functional groups can cause a very strong and irreversible inhibition of catalytic hydrogenation reactions over supported Ru[^23^, ^24^] and other metal catalysts.[^26^, ^27^] Furthermore, Mortensen et al.^[27]^ reported that chlorine‐containing organic molecules can act as catalyst poisons of supported metal catalysts, too.

Fatty acids may inhibit heterogeneously catalyzed reactions, for example, the aqueous‐phase reforming of glycerol over Pt/Al_2_O_3_,^[28]^ and the same has been shown for proteins, for example, in the hydrogenation of lactic acid over Ru/C (probably by pore blockage).^[24]^ On the other hand, residual glucose, sorbitol, succinic acid, propionic acid did not significantly influence the latter reaction system.^[24]^ Similarly, furfural or hydroxymethylfurfural in levulinic acid feedstock did not exhibit strong influence on the hydrogenation of levulinic acid.^[23]^ Formic acid, however, has a detrimental effect on the hydrogenation of levulinic acid as shown in several studies.[^23^, ^29^]

This overview clearly shows that in all cases additives or impurities have detrimental effects on the catalytic activity. Therefore, the influence of components in real effluents from biotechnological processes needs to be carefully investigated when combining them with heterogeneously catalyzed processing steps, such as the algae‐based photobiocatalytic GA production[^10^, ^11^, ^12^, ^13^] and the catalytic hydrogenation of the products solution to EG in the present study. Quantifying the respective influence of the different additives (acids/bases for pH regulation, inorganic salts, pH buffers and selected organic molecules of biological importance during the photobiocatalytic step) and identifying the main impediments for the direct hydrogenation of real algae‐derived GA solutions (AGAS) to EG are the central objective of this investigation. Most importantly, the feasibility of converting the sustainable photobiocatalytic feedstock GA into the important petrochemical platform chemical EG will be studied.

## Results and Discussion

### Composition of the Algal Medium

As a first step, the GA‐containing algal medium was analyzed to determine its composition and identify compounds that could potentially affect the catalytic hydrogenation reaction of GA to EG. Neither conversion of GA nor presence of any hydrogenated products was observed in preliminary experiments using as‐obtained AGAS and the same Ru/C catalyst and reaction conditions as in our previous study with GA model solutions.^[18]^ Therefore, the algal media must contain substances that are severely detrimental to the catalytic hydrogenation over the Ru/C catalyst.

The obtained AGAS used in this study contained GA in a concentration of about 20 mmol L^−1^ as quantification by both colorimetry and HPLC unambiguously shows. No considerable amounts of other organic side products were detected by GC and HPLC analysis. This confirms that the fixation and conversion of CO_2_ into GA is highly selective as described in the literature.[^10^, ^13^] Consequently, it was assumed that only the organic compounds added during the growth and production phase of the microalgae were present in the AGAS: (6‐Ethoxy‐2‐benzothiazole‐sulfonamide (EZA), which is used as a metabolic inhibitor, ethylenediaminetetraacetic acid (EDTA), applied as Fe^II^‐EDTA to facilitate Fe uptake by the algal cells, and two pH buffers (tris(hydroxymethyl)aminomethane (TRIS) and 2‐(*N*‐morpholino) ethanesulfonic acid (MES)). While the buffer concentrations (20–40 mmol L^−1^) are in the range of the GA concentration, EDTA and EZA are added in far lower amounts (0.025 and 0.050 mmol L^−1^, respectively). Considering the effects of heteroatom‐containing compounds like amino acids on similar catalytic reactions,[^23^, ^24^, ^26^] there is a high probability that these compounds, in particular sulfur‐containing EZA, can inhibit the conversion of GA to a certain degree.

Concerning inorganic additives, elemental analysis by ICP‐OES revealed the concentration of all relevant cations as well as the S and P content of the algal medium (Table S5). The amounts of most elements in the solution at the end of the photobiocatalytic experiment match the initial concentrations added to the algal medium very well. This confirms one key aspect of the concept of using algae as photobioreactors: Since the algal cells are kept in a GA production state where no new cell biomass is formed, there is hardly any observable nutrient consumption.[^10^, ^13^] In general, the concentration of all metals (except for K) is <1 mmol L^−1^, for most transition metals, even <0.02 mmol L^−1^. K is the element with the highest concentration in the AGAS (25 mmol L^−1^) and, most notably, its concentration is dramatically increased compared to the initially added nutrient salts containing K. However, KOH is used to neutralize the pH of the algal medium to compensate the gradual formation of GA during the photobiocatalytic CO_2_ fixation and, therefore, the K concentration corresponds well to the GA concentration in the algal medium (ca. 20 mmol L^−1^). It is known that alkali cations can poison catalytically active sites.[^25^, ^27^]

Furthermore, the solutions contain P (ca. 0.60 mmol L^−1^) and S (ca. 0.45 mmol L^−1^), most probably in the form of SO_4_^2−^ and HPO_4_^2−^ or H_2_PO_4_^−^, respectively. Sulfate is used as counter‐ion for most added cations, while phosphates are added as a key element for ion homeostasis in algal cultures. Since the elemental contents in the algal medium, in general, show little deviation from the amounts added during the biotechnological process, it can be assumed that the Cl concentration in the AGAS is also similar to the initial additive concentration around 7 mmol L^−1^, originating mostly from NH_4_Cl and CaCl_2_. Earlier studies showed that Cl can have inhibitory effects on hydrodeoxygenation reactions^[27]^ and that sulfate in the feed may poison supported metals used as hydrogenation catalysts.^[23]^

Finally, one of the most notable differences comparing aqueous model solutions and AGAS is the pH value. The pH value of a solution of 70 mmol L^−1^ GA in water was determined to be 2.5. On the other hand, the algal medium was kept at neutral pH to ensure optimal physiological performance for the algal cells. As mentioned above, this was done by gradually adding KOH to compensate the increasing concentration of GA. Studies on lactic acid hydrogenation showed that the reaction of calcium lactate only proceeds at sufficiently low pH since the reactant needs to be present in protonated form.[^19^, ^20^, ^24^] Therefore, the pH dependence of the catalytic hydrogenation of GA was investigated first, before assessing the influence of additive salts as well as the relevant organic compounds and eventually converting real AGAS.

### Hydrogenation of Model Solutions

**Figure 2:**
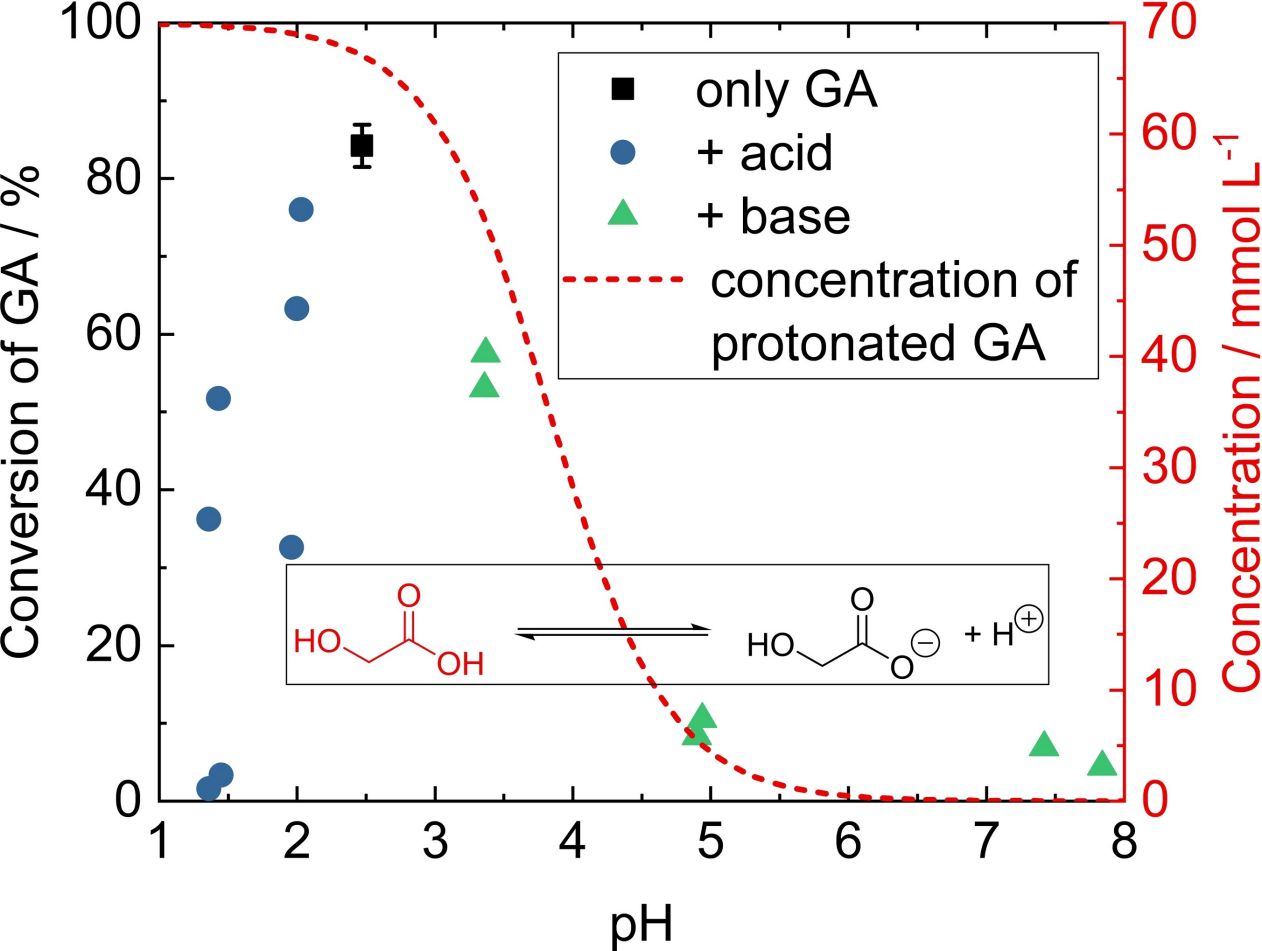
Conversion of GA over Ru/C as a function of pH. The pH of the model solution (only GA) was adjusted by addition of acids and bases. Shown in red is the pH‐dependent concentration of protonated GA calculated using the Henderson‐Hasselbalch equation. Standard deviation is based on five replications as outlined in Table S2. Reaction conditions: m_cat._=150 mg, V=80 mL, c_GA_=70 mmol L^−1^, t=4 h, *T*=150 °C, *p*_H2_=40 bar.

**Influence of pH value, acids and bases**. The pH dependence of the GA hydrogenation over Ru/C was investigated by adding different inorganic acids or bases to the model 70 mmol L^−1^ aqueous GA solution. This reference model solution shows a GA conversion of 84±3 %. The pH was varied from the initial value of about 2.5 (only 70 mmol L^−1^ GA in water) up to neutral pH (ca. 7–8 as in the neutralized algal medium) and down to 1.4. The results are shown in Figure 2 and listed in Table S3.

Increasing pH results in gradually lower GA conversion, down to about 55 % at pH 3.4 and ≤10 % at pH ≥4.9. This behavior is in good agreement with the results reported by Zhang et al.^[20]^ and Binczarski et al.,^[19]^ who observed that calcium lactate needs to be acidified with H_2_SO_4_ to be converted into propylene glycol. Optimum pH was found to be in the range of 2.5–3.0. In the latter study, the authors suggested that this is due to formation of increasing amounts of free lactate ions caused by the precipitation of CaSO_4_.^[19]^ These free lactate ions, as opposed to calcium lactate, can then be converted into propylene glycol. On the other hand, Zhang et al.^[20]^ explained their similar findings of pH influence on the lactic acid hydrogenation by higher amounts of protonated lactic acid compared to lactate ions, and suggested that mainly the protonated form of lactic acid can undergo catalytic hydrogenation. By adding KOH to a lactic acid solution, they showed that there is a pH dependence similar to the one observed in this study. Besides pH‐dependent conversion, Figure 2 also depicts the calculated concentration of protonated GA over the pH range. A clear correlation between decreasing concentration of protonated GA and deceasing conversion is apparent from 2.4 to neutral pH. This affirms the assumption by Zhang et al.^[20]^ that GA has to be protonated to undergo reaction.

**Figure 3:**
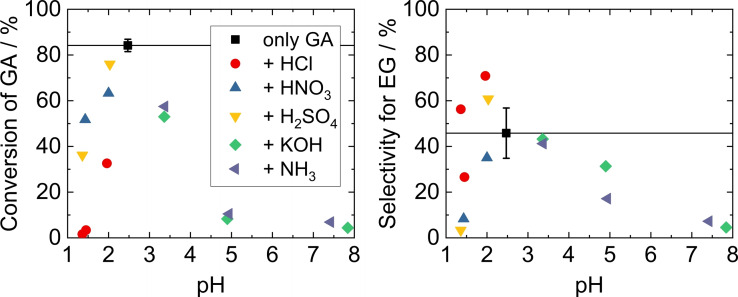
GA conversion (left) and selectivity for EG (right) in the hydrogenation of GA over Ru/C, for different pH values. The pH of the model solution (only GA) was adjusted by addition of different acids and bases. The added concentrations are listed in Table S3. Standard deviations are based on five replications as outlined in Table S2. Reaction conditions: m_cat._=150 mg, V=80 mL, c_GA_=70 mmol L^−1^, t=4 h, *T*=150 °C, *p*_H2_=40 bar.

Previous studies only investigated pH values as low as 2.0. In this study, we used different inorganic acids to further lower the pH to 1.4 (Figure 2). Regardless of the acid used, GA conversion is significantly decreased even at pH 2.0, which indicates that the pH of the model solution (ca. 2.5) allows for the highest GA conversion. The equilibrium between protonated GA and deprotonated glycolate does not offer an explanation for the low catalytic activity in the low pH region, since >95 % of the reactant can be assumed to be in the reactive protonated GA form. Furthermore, in contrast to increasing pH where no significant difference between using KOH or NH_3_ was found, there is a strong dependence on the respective acid used for lowering the pH, which is shown in detail in Figure 3.

It is apparent that aqueous HCl leads to a much stronger inhibition of GA conversion compared to HNO_3_ or H_2_SO_4_ at comparable pH. While for the latter two still around 50 and 35 % GA conversion was observed at pH 1.4, practically no GA was converted when adding HCl to obtain the same pH. The differences between the three acids are most likely caused by their respective anions since the proton concentration is comparable in all cases. The different acids also influence the selectivity for EG in the reaction. Selectivity for EG was strongly decreased for HNO_3_ and H_2_SO_4_ (<10 % at pH 1.4 compared to 46 % with only GA model solution at pH 2.5). Similar to our previous study,^[18]^ only small amounts of acetic acid (in all cases <10 % yield) were formed as a product besides EG, leading to the assumption that mostly gaseous products like methane and ethane were formed, which were not quantified. It should be noted that the carbon balance can be closed at appropriate conditions, and up to 90 % EG yield are possible.^[18]^

The direct influence of the pH value becomes even more obvious when comparing the effect of the different acids (HCl, HNO_3_, H_2_SO_4_) on the GA model solution to the impact of their corresponding Na salts. At the same anion concentration, the salts only marginally impact the pH of the solution (Table S3). Adding NaCl in a concentration of 42 mmol L^−1^ results in a GA conversion of 29 % (at pH 2.4, as compared to 84 % using the model GA solution with a pH of 2.5) while 42 mmol L^−1^ HCl decreases pH to 1.4 and conversion to about 2 %. Thus, regardless of any influence of the anion, the proton concentration, that is, the pH of the reaction solution, has its own detrimental influence on the catalytic reaction. The same applies to H_2_SO_4_, which in a concentration of 35 mmol L^−1^ reduces pH to 1.4 and GA conversion to 36 %, and Na_2_SO_4_, in the presence of which a significantly higher conversion of 44 % was reached even at a concentration of 100 mmol L^−1^ (at a pH of ca. 2.6).

A possible reason for the lower catalytic activity caused by the low pH could be structural disintegration of the Ru/C catalyst. While the stability of the support material was not investigated under reaction conditions, elemental analysis by ICP‐OES of solutions after 4 h of reaction (Table S6) reveals that, even at pH 1.4, less than 1 % of Ru was dissolved from the catalyst into the reaction medium. This makes Ru leaching an unlikely cause of low catalytic activity. On the other hand, H^+^ may also directly alter the interactions between the Ru surface, GA and water. Along these lines, water molecules have been reported to play a crucial role in enabling high catalytic activity of Ru catalysts.^[30]^

Summarizing the influence of pH, two effects result in an optimal pH of the reaction medium at about 2.5. At higher pH values, GA is deprotonated to glycolate ions. Consistent with literature reports,^[20]^ it is suggested that deprotonated GA does not undergo hydrogenation. At lower pH, the reaction is inhibited due to reasons that are still unclear. As a consequence, AGAS (and, similarly, also fermentation‐derived GA broths), in which GA is present as deprotonated glycolate ions due to the pH regulation during the biotechnological process, must be acidified to allow for catalytic hydrogenation to take place. The findings indicate that an optimal pH value exists and over‐acidification is detrimental to hydrogenation activity. However, acidification also increases the concentration of inorganic ions in the reaction solution, whose influence is discussed in the subsequent section.

**Figure 4:**
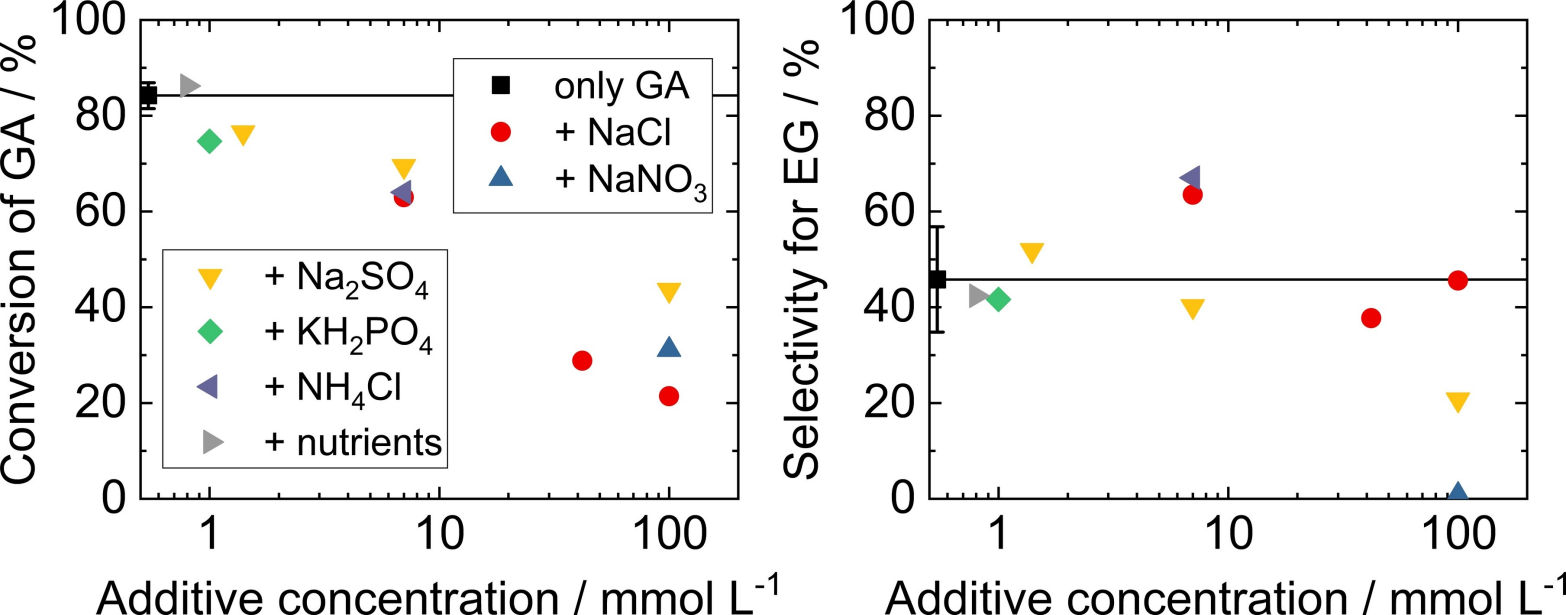
Conversion of GA (left) and selectivity for EG (right) in the hydrogenation of GA model solutions over Ru/C with different inorganic salt additives at varying concentrations. Detailed values are given in Table S3. Standard deviations are based on five replications as outlined in Table S2. Reaction conditions: m_cat._=150 mg, V=80 mL, c_GA_=70 mmol L^−1^, t=4 h, *T*=150 °C, *p*_H2_=40 bar.

**Influence of inorganic salts**. An overview of all catalytic results from experiments with additional inorganic salts (NaCl, Na_2_SO_4_, NaNO_3_, NH_4_Cl, K_2_HPO_4_) in the GA model solution is shown in Figure 4. Inorganic salts are present in the algal medium as nutrients and are formed as by‐products of the acidification of the pH‐controlled medium.

Acidification of neutral AGAS with HCl, HNO_3_ and H_2_SO_4_ results in the presence of comparatively large concentrations of their anions in the range of the GA concentration (70 mmol L^−1^). Therefore, experiments with up to 100 mmol L^−1^ of the corresponding Na salts were conducted. Comparing GA conversion at different salt concentrations, it is apparent that higher concentrations of any additive salt result in more pronounced decrease in catalytic activity. It is well known that additional ions in aqueous solutions result in a “salting‐out” effect, that is, lowering the solubility of gases in the liquid phase. In a previous study by Zhang et al.,^[20]^ lower lactic acid hydrogenation activity over a similar Ru/C catalyst in the presence of increasing K_2_SO_4_ concentrations was suggested to be caused by this effect of reduced H_2_ solubility.

Besides this general influence, comparison of NaCl, NaNO_3_ and Na_2_SO_4_ at 100 mmol L^−1^ additive salt concentration reveals that Cl^−^ has the strongest negative effect on GA conversion, a trend that is also confirmed at a lower concentration of 7 mmol L^−1^. At 100 mmol L^−1^ salt concentration, GA conversion is lowered to 21 % when NaCl is used, to 31 % when NaNO_3_ and to 44 % when Na_2_SO_4_ is present, as shown in Figure 4. Therefore, it can be concluded that the catalytic hydrogenation reaction is least inhibited by the presence of sulfate. Regarding the selectivity of the catalytic hydrogenation reaction, Cl^−^ ions do not significantly influence the selectivity for the target product EG. On the other hand, in the presence of 100 mmol L^−1^ nitrate ions, no EG was formed despite a conversion of 31 %. Since nitrate is known to have an affinity to supported metal catalysts and can undergo catalytic decomposition,^[31]^ it is possible that nitrate interacts with the catalytically active sites of the hydrogenation reaction in a more pronounced way than the other two ions. However, it is unlikely that any of the three anions chloride, sulfate or nitrate acts as a strong and selective poison to the Ru/C catalyst in the concentration range tested here. Despite a more than hundred‐fold excess acid/Ru (molar ratio), catalytic activity is far from completely suppressed. This is in line with studies by Zhang et al.,[^20^, ^24^] who did not find a poisoning effect of sulfate or phosphate ions on the heterogeneously catalyzed hydrogenation of lactic acid over Ru/C. Instead, they suggested contributions of pH change as well as lower H_2_ solubility to negatively affect catalytic activity.^[20]^ Differences observed between different anions could then, at least in part, be a result of the strength of the salting‐out effect of the different ions.^[32]^ However, a more direct deactivating interaction with the catalyst can also not be excluded. This may be the case in the unexpected strong detrimental effect of nitrate on the selectivity of the hydrogenation reaction and in case of the particularly strong decrease in activity in presence of chloride.

Besides the three salts NaCl, NaNO_3_ and Na_2_SO_4_ that are present in the algal medium after acidification with the respective acid, there is a variety of salts added in different concentrations during the photobiocatalytic step. The detailed composition was discussed above and is also listed in Table S5. The additive salt with the highest concentration (7.0 mmol L^−1^) in the initial algal medium is NH_4_Cl. Its presence in the reaction medium decreases the catalytic activity by 25 %, as shown in Figure 4. Comparing this to the previously mentioned NaCl and Na_2_SO_4_ solutions of the same concentration of 7.0 mmol L^−1^ allows an assessment as to whether mainly Cl^−^ or the pseudo‐alkali ion NH_4_^+^ causes this decrease in catalytic hydrogenation activity. While GA conversion in the presence of NaCl was comparable to that in presence of NH_4_Cl (63 % GA conversion), it only dropped to 77 % for Na_2_SO_4_ addition despite a correspondingly doubled Na^+^ concentration. Therefore, Cl^−^ must be considered the main inhibiting ion contained in these salts, which also matches the results of the pH variation shown above.

The concentrations of most other salts in the algal medium is considerably lower. Therefore, eight salts were added at once to the GA model solution (the detailed composition is shown in Table S5). This salt mixture is referred to as nutrients due to their biological function in the photobiocatalytic GA production step. Despite the presence of transition metals (Cu, Co, Ni, Zn, Mn, Mo), the catalytic activity and selectivity remained the same as for the additive‐free model solution within the uncertainty of the experiment, as shown in Figure 4. Similarly, the addition of 1.0 mmol L^−1^ phosphate or 1.4 mmol L^−1^ sulfate showed only a small influence (i. e., a decrease in GA conversion from 84 % to ca. 77 % and 79 %) on the heterogeneously catalyzed hydrogenation of GA over the Ru/C catalyst. Note that, despite their very low concentration of in total <1 mmol L^−1^, the nutrient salts are still present in excess compared to the number of Ru atoms on the catalyst (equivalent to 0.6 mmol L^−1^) and could therefore significantly poison the catalyst. However, the results indicate that no strong deactivating interaction between the nutrient salts and the Ru catalyst exist and that the nutrient salt mixture is tolerated in the aqueous GA feed.

Based on the investigation of inorganic salt additives, two conclusions can be drawn for the use of algae‐derived GA‐containing feedstock. At low concentrations (up to 7 mmol L^−1^), all relevant inorganic salts in the algal medium have no considerable effect on the on the catalytic hydrogenation reaction of GA over Ru/C in the batch reactor. However, the contribution of acidification of the algal medium has to be considered. Since a neutral pH is necessary during the algae‐based GA synthesis, but GA needing to exist in the protonated form at a pH of about 2.5 for effective hydrogenation, acidification is crucially required before the catalytic conversion. This causes ion concentrations in the range of 100 mmol L^−1^ of the anions of the added mineral acid. Their influence was assessed by adding the respective Na salts, among which Na_2_SO_4_ shows the least inhibition of catalytic activity. Therefore, the corresponding acid H_2_SO_4_ is considered the most suitable choice for acidification of the algal medium before the catalytic reaction. An optimization of the algal medium for an improved compatibility with the demands of heterogeneous catalysis appears possible, for example, by omitting ammonium ions or reducing the concentration of chloride.

**Figure 5:**
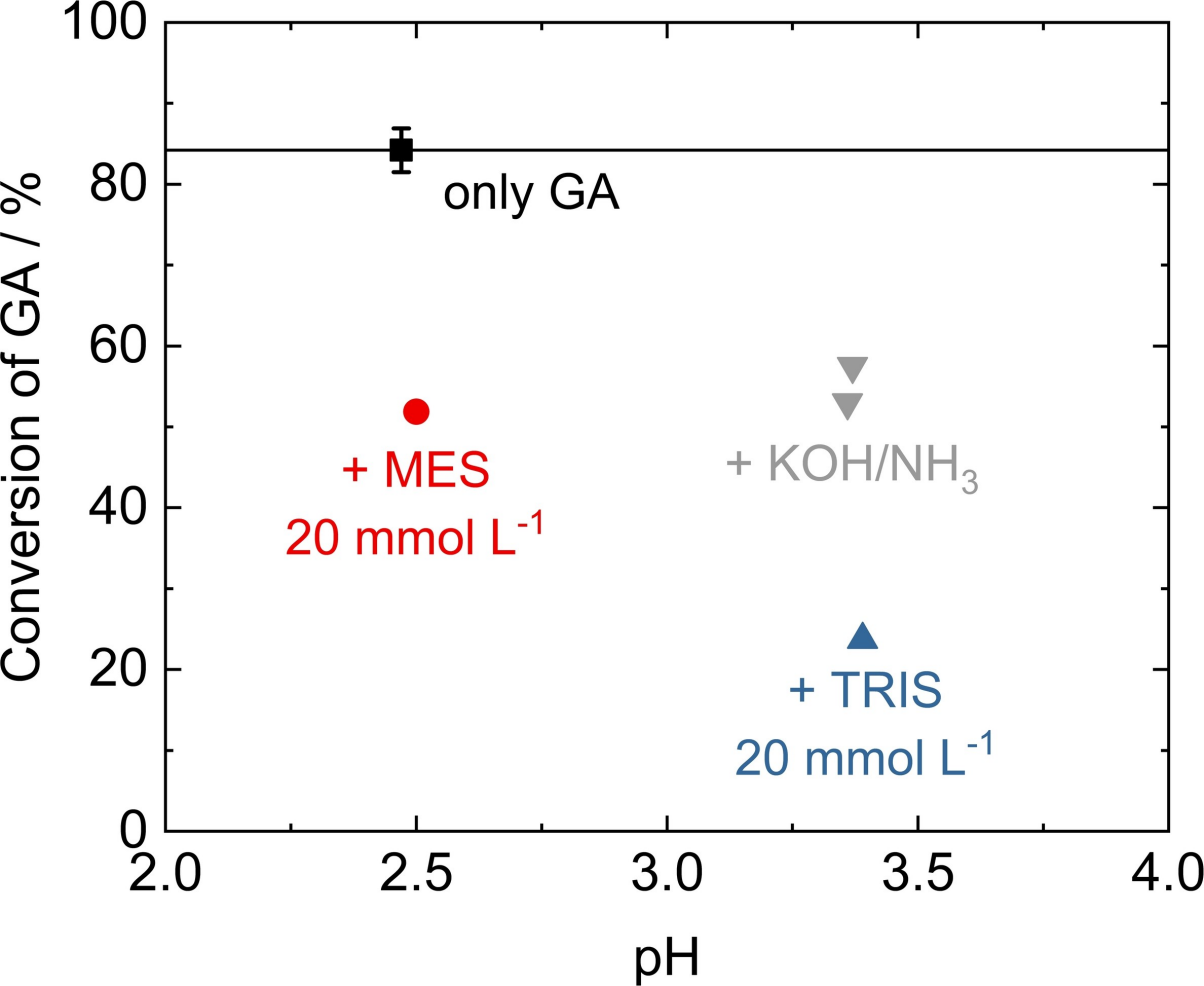
Conversion of GA as a function of pH in the hydrogenation of GA model solutions over Ru/C after addition of TRIS or MES pH buffer. Model solutions with adjusted pH by addition of KOH or NH_3_ are included for comparison. Standard deviation is based on five replications as outlined in Table S2. Reaction conditions: m_cat._=150 mg, V=80 mL, c_GA_=70 mmol L^−1^, t=4 h, *T*=150 °C, *p*_H2_=40 bar.

**Influence of pH buffers**. To maintain a physiological pH value during algae‐based GA synthesis in this study, either TRIS, which has been used previously,^[11]^ or MES were added as buffer to the algal medium during GA production. The influence of the respective buffer on the successive GA hydrogenation over Ru/C was therefore investigated. The results, summarized in Figure 5, show that both MES and TRIS negatively affect the catalytic conversion of GA. Addition of 20 mmol L^−1^ MES to the model solution, the amount present in the algal medium during the photobiocatalytic GA production step, results in a decrease in GA conversion from 84 % to 52 %. The same amount of TRIS leads to a decreased conversion of 24 %. Due to the excess amounts of GA, the buffers are clearly not in their effective buffer pH range in case of the model solution. The pH value remains almost unchanged upon addition of MES (ca. 2.5), therefore, in this case, other factors must be responsible for the inhibitory effect. However, addition of TRIS increased the pH to about 3.4, which alone can be expected to result in lower conversion due to the deprotonation of GA, as has been discussed above. However, when comparing the TRIS‐containing GA model solution to model solutions with KOH or NH_3_ at similar pH (GA conversion ca. 55 %), it becomes apparent that also for TRIS other effects than the change in pH are responsible for the decrease. At the same time, selectivity for EG was decreased, even though not significantly (Figure S1).

The molecules from both buffers contain a nitrogen heteroatom in the form of an amino group, and MES has an additional sulfur heteroatom in the form of a sulfonic acid group. It is well known that amino acids can interfere with other catalytic hydrogenation reactions.[^23^, ^24^, ^26^] In all studies, molecules containing only nitrogen heteroatoms had a considerably less pronounced inhibitory effect than sulfur‐containing ones. Zhang et al.^[24]^ showed that the inhibition by alanine is reversible, indicating that it does not act as an irreversible catalyst poison. However, it is assumed to preferentially adsorb on the active metal sites of the catalyst. A similar influence of MES and TRIS appears likely. Even though MES contains a sulfur atom in the sulfonic acid group, it did not show a strong effect associated with selective inhibition, as was described for other sulfur containing functional groups, for example, by Schwartz et al.^[26]^ Similar to sulfate, which also showed no indications for selective and irreversible poisoning, the higher oxidation state of sulfur in the sulfonic acid group appears to drastically reduce the potential of the sulfur atom to strongly bind to the active metal site as compared to the thiols, thioethers or S‐containing heterocycles reported by Schwartz et al.^[26]^

In summary, both tested buffers showed strong inhibitory effects, resulting in a decrease in GA conversion of about 50 % after 4 h of reaction. This does not take into account pH effects, which can be resolved by acidification. While strong catalyst poisoning was found to be unlikely, the presence of buffers significantly decreases catalytic activity. The most critical point is the comparatively high concentration of the buffers needed to balance the biological system, especially under high concentrations of accumulating GA. Considering the use of algal‐produced GA as feedstock for the catalytic hydrogenation reaction, the overall process would benefit from reducing the amounts of buffer used in the biotechnological step if physiologically feasible.

**Figure 6:**
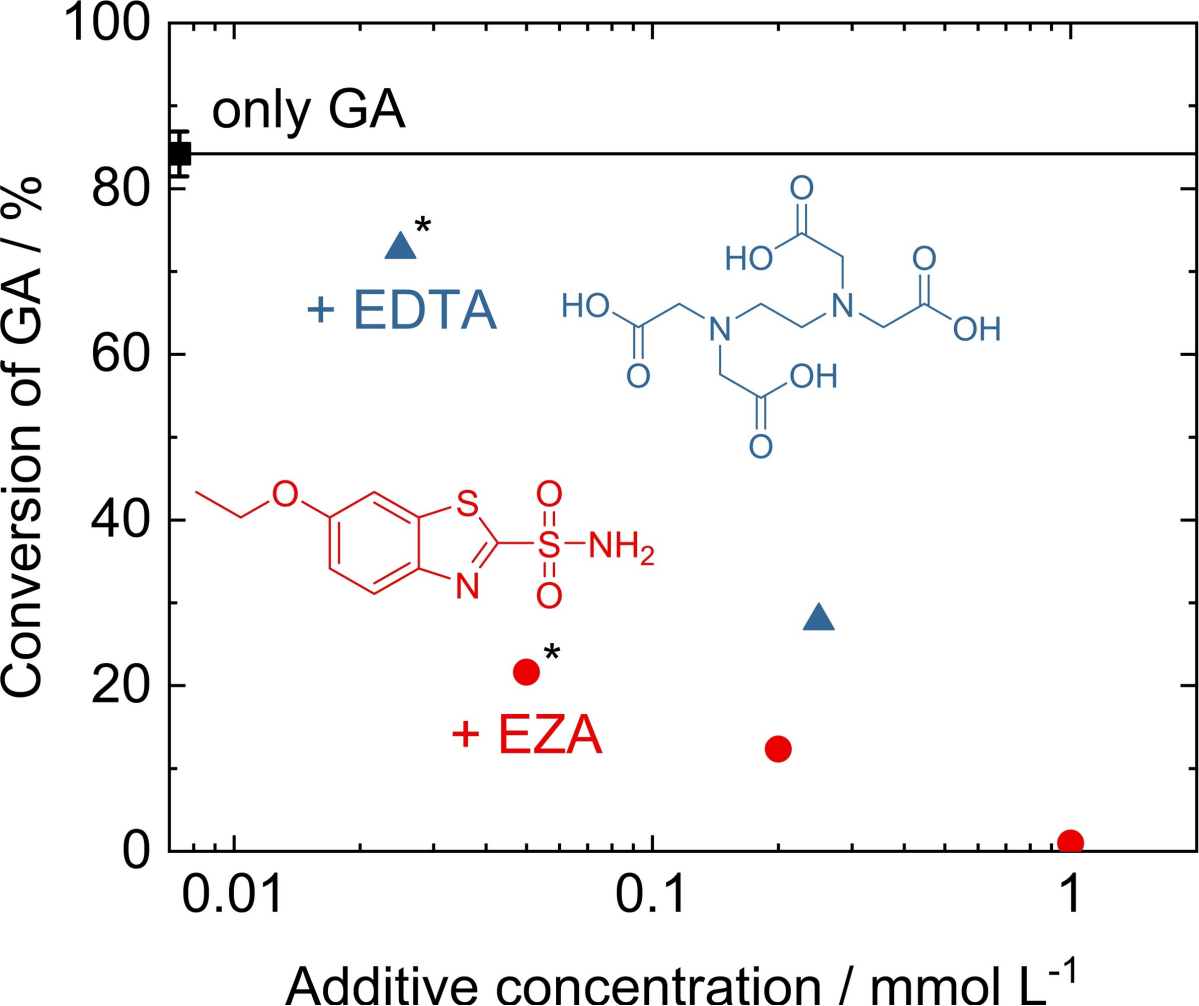
Conversion of GA in the hydrogenation of GA model solutions over Ru/C after addition of EDTA or metabolic inhibitor EZA in different concentrations. (*) denotes the concentration present in the algal medium. Standard deviation are based on five replications as outlined in Table S2. Reaction conditions: m_cat._=150 mg, V=80 mL, c_GA_=70 mmol L^−1^, t=4 h, *T*=150 °C, *p*_H2_=40 bar.

**Influence of complexing agent and metabolic inhibitor**. Besides the pH buffers, two other organic compounds were added to the algal media: the complexing agent EDTA and a metabolic inhibitor EZA. The concentration of both EDTA and EZA is ca. 1000 times lower than those of the buffers. Nevertheless, they have considerable influence on the conversion of GA in the catalytic hydrogenation over Ru/C (Figure 6). At the same time, no significant changes in selectivity for EG are observed (Figure S2).

Addition of 0.025 mmol L^−1^ Fe‐EDTA, which is the concentration present in the algal medium, leads to a slight but significant drop in GA conversion from 84 % (no additive) to 73 %. At tenfold concentration (0.25 mmol L^−1^), however, GA conversion further decreases to 28 %. Therefore, EDTA is clearly more potent in inhibiting the catalytic reaction compared to, for example, TRIS. The fact that EDTA is a larger molecule containing two amino groups as well as multiple carboxylic groups could explain this stronger effect of active site blocking. 150 mg catalyst contain 0.068 mmol Ru, of which 0.022 mmol are surface atoms (Ru dispersion 32 %). Assuming this, 80 mL of 0.25 mmol L^−1^ EDTA contain enough molecules (0.020 mmol) to bind to almost all surface Ru atoms given a 1 : 1 binding stoichiometry. Interestingly, when 0.25 mmol L^−1^ EDTA were added to the reaction solution, an unusually high amount of acetic acid was found in the product solution, which equates to a selectivity for acetic acid of >30 % (compared to ca. 9 % in the reference reaction). Since, at the same time, EG selectivity is comparable to the reference experiment, it is more likely that EDTA is decomposed under reaction conditions to form acetic acid, as has been observed previously under hydrothermal conditions,^[33]^ than a direct effect of EDTA on the selectivity of the catalytic reaction. Furthermore, it can be speculated if GA hydrogenation is less inhibited after EDTA is (partially) decomposed.

Overall, EDTA has the potential to inhibit the catalytic reaction strongly. However, the low concentration in the algal medium results in only a slight decrease in catalytic activity in the batch reaction. On the other hand, in continuous flow experiments there might be a risk of accumulation of EDTA in the reactor and a complete inhibition of the reaction.

EZA is the only organic compound with a sulfur‐containing heterocycle in the algal medium. In an application‐relevant concentration of 0.050 mmol L^−1^, EZA drastically reduces catalytic activity (GA conversion 22 %) as shown in Figure 6. Therefore, EZA must interact even more strongly with the catalyst than EDTA. Note that in the typical concentration of 0.050 mmol L^−1^, the ratio between surface Ru atoms and EZA is around 4 : 1. Further increasing the EZA concentration up to an excess amount of 1.0 mmol L^−1^ (an amount which is already beyond its solubility limit at room temperature) eventually leads to a complete inhibition of the GA hydrogenation reaction. It is known that sulfur‐containing biogenic organic compounds can strongly poison noble metal catalysts.[^20^, ^23^, ^24^, ^26^] Especially the study by Zhang et al.^[24]^ clearly proves the irreversible nature of the poisoning probably caused by strong interaction of the molecules with the active Ru sites of the catalyst. As mentioned above, compounds containing sulfur in higher oxidation states have a far lower impact on the reaction and probably cannot irreversibly poison the catalyst as EZA or compounds like thiols.

The inhibitory effect of EZA with regard to the application of algae‐derived GA is most evident from comparing the model solution containing only 0.050 mmol L^−1^ additional EZA to a reaction solution containing all other additive salts, EDTA and TRIS buffer (Table S3, entry 31, pH adjusted to 2.5). EZA alone decreases GA conversion to 22 %, while the combined effect of all other compounds results in a comparable decrease to 25 %. Therefore, EZA is by far the compound with the strongest inhibitory effect and should preferably not be present at all in the reaction medium. Besides the EZA‐based GA production approach^[11]^ used to produce AGAS 1, new biotechnological concepts might be required, such as the modified approach included in this study, which provided EZA‐free AGAS 2.

### Catalytic Hydrogenation of Algae‐Derived Glycolic Acid Solutions

The investigation of the influence of pH on the catalyzed hydrogenation reaction showed that GA‐containing algae media need to be acidified to be generate GA in the protonated form. This matches the results of preliminary experiments indicating that GA in as‐obtained AGAS could not be converted into EG without prior modifications. H_2_SO_4_ was used to acidify the AGAS to pH 2.5, which is better suited for this purpose than HCl or HNO_3_ according to the results shown above. Two AGAS (both acidified by this procedure before the reaction) were employed as reactant solutions. AGAS 1 was produced by the previously published approach^[11]^ and AGAS 2 derived by a novel approach,^[34]^ which does not require the metabolic inhibitor EZA.

**Figure 7:**
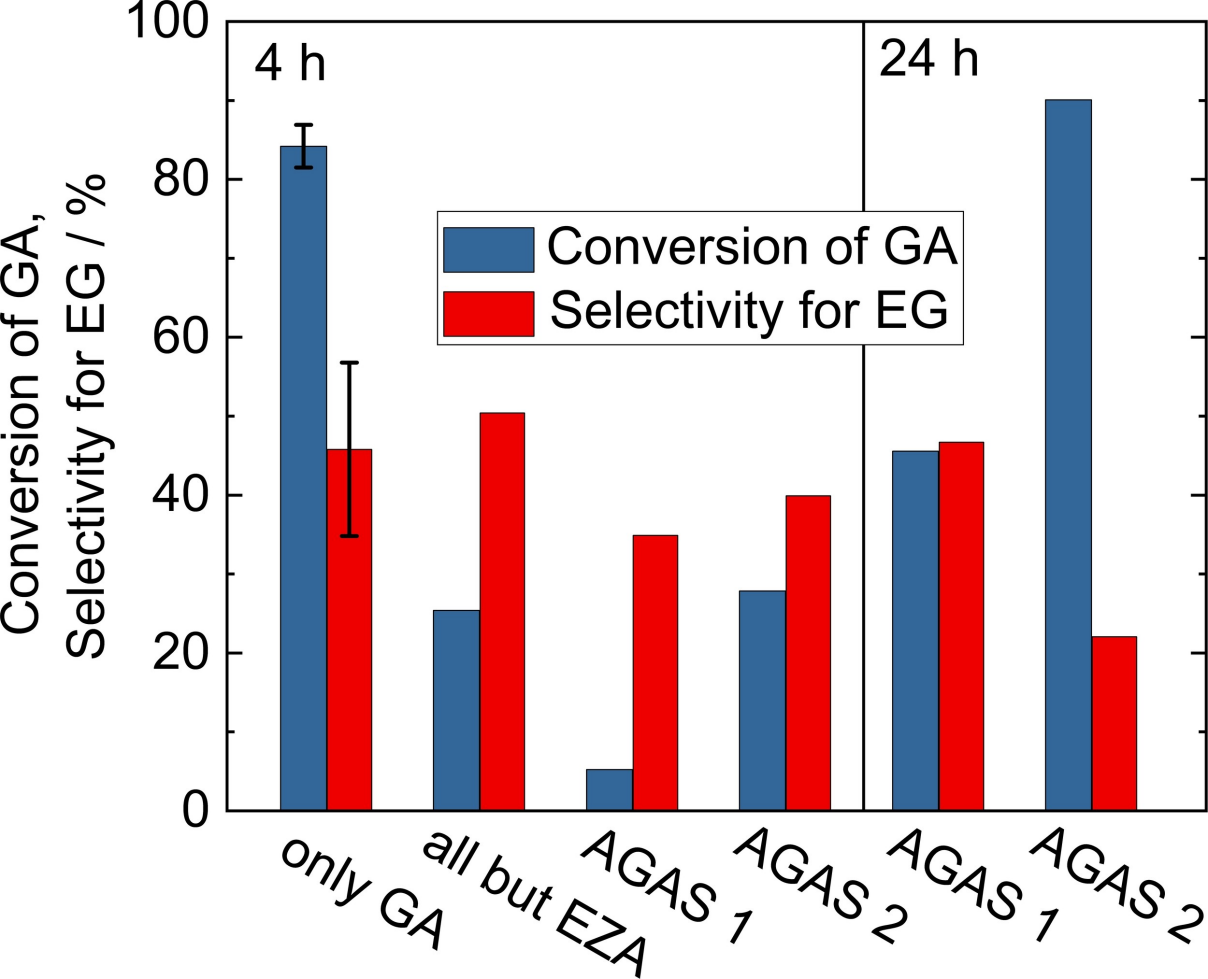
Conversion of GA and selectivity for EG in the hydrogenation of GA over Ru/C using model reactant solutions or acidified algae‐derived GA solutions (AGAS 1 and 2). Detailed values in Table S3, composition of algal solutions in Table S4. Standard deviations are based on five replications as outlined in Table S2. Reaction conditions: m_cat._=150 mg, V=80 mL, c_GA_=70 mmol L^−1^, t=4 h, *T*=150 °C, *p*_H2_=40 bar.

The results displayed in Figure 7 clearly show that the acidification strategy is successful. After 4 h, the typical reaction time of the experiments presented above, GA conversion is observed for both AGAS 1 and 2. As expected, GA conversions are considerably lower than for the GA model solution without additives (84 %). Furthermore, conversion of GA in AGAS 1 (5 %) was about five times lower than of AGAS 2 (28 %). This is another clear indication of the inhibitory influence of EZA, which probably acts as a catalyst poison. Furthermore, AGAS 2, which did not contain EZA, gave results comparable to a model GA solution containing all additives (salts, TRIS buffer, EDTA) but EZA (ca. 26 % GA conversion). This agreement of the combined inhibitory effect of all additives investigated in the previous sections and the real AGAS 2 shows that all relevant compounds were included in the study. This also indicates that the formation of significant amounts of other organic compounds during the photobiocatalytic GA production step is unlikely.

One way of increasing GA conversion is to prolong the reaction time. After 24 h, GA conversion reached 46 % (AGAS 1) and 90 % (AGAS 2). For the latter, however, selectivity for EG decreased from 40 % (after 4 h) to 22 %, which could be an indication of decomposition of EG towards the end of the reaction time. This was confirmed by investigating the stability of a 70 mmol L^−1^ EG solution at reaction conditions in the presence of the Ru/C catalyst, which showed that after 4 h more than 70 % of EG was converted into ethanol and gas‐phase products. It should be noted that the reaction conditions are not optimized for the algal reaction medium and, consequently, the EG yield does not exceed 21 %. However, as reported in our previous study,^[18]^ EG selectivity can be significantly increased up to almost quantitative EG formation at lower reaction temperature (e. g., 105 °C), which also leads to a closure of the carbon balance. However, in case of AGAS, this would probably come at the cost of even longer reaction times. As a consequence, better catalysts are required that provide higher selectivity or are highly active at lower temperatures (<120 °C) to benefit from the increased selectivity at lower temperature.

The experiments with acidified AGAS show that the conversion of GA in such media into EG is possible by heterogeneous catalysis. The reaction tolerates all additives and impurities, however at drastically reduced catalytic activity.

## Conclusion

A novel two step‐process was successfully employed to first form GA from CO_2_ and sunlight by microalgae in a photobiocatalytic step, and subsequently convert GA accumulated in the algal medium in a second, heterogeneously catalyzed hydrogenation step over a Ru/C catalyst into EG. A systematic investigation into the influence of different biologically relevant additives on the hydrogenation step revealed that the pH value of the algea‐derived GA solution (AGAS) needs to be adjusted by acidification, preferably with H_2_SO_4_. While inorganic salts only slightly decrease the catalytic hydrogenation activity, heteroatom‐containing organic molecules, such as the pH buffers TRIS and MES as well as EDTA, have a considerably stronger detrimental effect. In particular, sulfur‐containing EZA, used as a metabolic inhibitor in the biotechnological process, has the strongest deactivating effect on the catalytic reaction, most likely due to poisoning of the active Ru sites on the catalyst. These findings are expected to be relevant for similar heterogeneously catalyzed biomass valorization processes.

Catalytic experiments with real AGAS after acidification proved that the overall two‐step concept consisting of GA formation using microalgae and subsequent direct hydrogenation EG is feasible. The catalytic conversion takes place in the presence of all additives and impurities from the current biotechnological process, although inhibition cannot be avoided completely. EG yields of up to 21 % were obtained under non‐optimized reaction conditions. Overall, the presented two‐stage approach presents a promising route to sustainable EG from CO_2_ and sunlight as an alternative to conventional biomass utilization. Future research should be directed towards a continuous hydrogenation process, which will not only allow direct coupling of the two catalytic steps, but would also provide insight into the reversibility of catalyst poisoning and catalyst deactivation over repeated use and longer time‐on‐stream. Moreover, strategies to remove impurities should be investigated to mitigate the inhibitory effect on the catalytic reaction, which is also detrimental from an economic point of view.

## Experimental Section

**Culture conditions during the photobiocatalytic step**. Algae‐derived GA solution 1 (AGAS 1): Cells of *Chlamydomonas reinhardtii* (SAG 11–32b, Culture Collection of Algae, Göttingen, Germany) were grown with modified TAP medium in a 400 mL flat panel photobioreactor (FMT 150, PSI, Drasov, Czech Republic) as a continuous culture according to Taubert et al.^[11]^ Optical density was adjusted to correspond to chlorophyll a contents of 2‐3 mg L^−1^ (low biomass) and 8‐9 mg L^−1^ (high biomass). Temperature gradients (15‐30 °C) and dynamic light conditions (1700 μmol photons m^−2^ s^−1^ maximum irradiance, 14/10 h light/dark) were applied to simulate outdoor conditions in a temperate zone. GA production was induced by changing the O_2_/CO_2_ ratio of aeration from biomass producing conditions (21 %/5 %) to photorespiratory conditions (40 %/0.2 %). At the same time, 50 μmol L^−1^ of the inhibitor EZA (6‐ethoxy‐2‐benzothiazolesulfonamide; Sigma Aldrich, Darmstadt, Germany) was added to the medium to induce photorespiratory conditions by preventing the induction of carbon concentrating mechanisms (CCM). Without this inhibition the cells become insensitive against external O_2_/CO_2_ ratios and the cells grow instead of producing GA. The culture was harvested after 7–21 days, depending on the achieved GA concentration in the medium (13‐20 mmol L^−1^). For further details see Taubert et al.^[11]^ The obtained cell free medium was sterile filtered (PES, 0.2 μm pore size) and stored at −20 °C until further usage.

AGAS 2: A *Chlamydomonas reinhardtii* mutant strain (CC‐5759D6 cia5/gyd, Chlamydomonas Resource Center, Minnesota, USA) defective in CCM and glycolate dehydrogenase (GYD), which produces GA under ambient air without the addition of EZA, was used to minimize effects of EZA. The CC‐5759D6 cia5/gyd strain was obtained from conventional crossing of the two strains LMJ.SG0182.017965 (gene cassette disrupting the GYD gene) x CC‐2702 (CCM master regulator cia5 point mutation knock out). Both strains were crossed with wild type (WT) strains to improve fitness; SAG 11–32b (WT) x LMJ.SG0182.017965 and CC‐410 (WT) x CC‐2702 (cia5).^[34]^ All strains were acquired from the Clip‐Library (Chlamydomonas Resource Centre, Minnesota, USA) except strain SAG 11–32b (Culture Collection of Algae, Gottingen, Germany). The CC‐5759D6 cia5/gyd strain was cultivated in batch culture for about 11 days. Cultivation was performed with block light (150 μmol photons m^−2^ s^−1^, 14/10 h light/dark) in modified TAP medium as mentioned above. The TRIS buffer was replaced by 20 mmol L^−1^ MES (2‐(*N*‐morpholino)ethanesulfonic acid). For CO_2_ supply, 38 mmol L^−1^ carbonate was added to the media according to Pörs et al.^[35]^ The cells were harvested in log phase with a chlorophyll a content of >2 mg L^−1^. The obtained cell free medium was sterile filtered (PES membrane, 0.2 μm pore size) for further use.

**Analysis of the algal medium**. The GA concentrations in the algal media were quantified using a colorimetric method.^[36]^ For derivatization, 50 μL of the GA containing algal medium was dissolved in 1.5 mL of concentrated H_2_SO_4_ containing 2,7‐dihydroxynaphthalene (0.01 %) and heated to 100 °C for 20 min. After rapid cooling the absorbance of the samples was measured at 540 nm using a Specord M250 spectrometer from Zeiss. In addition, samples were analyzed using gas chromatography (GC) and high‐performance liquid chromatography (HPLC) as described below.

The elemental contents of several metal cations as well as the S and P in the algal media were determined by optical emission spectroscopy with inductively coupled plasma (ICP‐OES). A Perkin Elmer Optima 8000 instrument equipped with a peristaltic pump was used. Three differently diluted solutions (dilution ratios 1 : 1, 1 : 15, 1 : 50) were prepared from each sample and subsequently analyzed. The following elements were identified using the corresponding wavelength in brackets: Ca (317.933 nm), Co (238.892 nm), Cu (327.393 nm), Fe (259.939 nm), K (766.490 nm), Mg (285.213 nm), Mo (203.845 nm), Na (589.592 nm), P (213.617 nm), S (180.669 nm) and Zn (206.200 nm). Quantification of the respective elemental contents was based on external calibrations for each element using commercially available standard solutions.

The pH value of aqueous solutions was determined with a Mettler Toledo SevenCompact Duo instrument connected to an InLab Routine Pro pH electrode. Prior to measurements, the equipment was calibrated using commercially available buffer solutions.

**Heterogeneously catalyzed hydrogenation of glycolic acid**. For all experiments, a commercially available carbon‐supported Ru catalyst with a nominal Ru loading of 5 wt.% was used (Ru/C, ABCR). The same catalyst was already used in our previous study containing detailed characterization data and information on the corresponding characterization methods.^[18]^ An overview of the key material properties (textural properties and metal dispersion) is shown in Table S1. Ru/C was reduced directly before every catalytic experiment in a tubular furnace. For this, the catalyst was heated to 250 °C in a flow of H_2_ (5 mL min^−1^) and N_2_ (20 mL min^−1^) and kept at that temperature for 2 h.

Aqueous‐phase hydrogenation experiments of GA were conducted in a batch autoclave reactor (Berghof BR‐200) with a 200 mL Teflon liner. The autoclave was placed inside a heating mantle mounted on top of a hot plate magnetic stirrer. The setup included a dip tube for liquid‐phase sampling as well as for N_2_ and H_2_ gas supply.

All catalytic experiments were conducted as follows: 80 mL of GA‐containing reaction mixture (either model mixtures or AGAS, see following sections) and 150 mg of pre‐reduced Ru/C were filled into the reactor. The reactor was purged with N_2_ and heated up to 150 °C. To start the hydrogenation reaction, 40 bar H_2_ pressure was applied to the autoclave. After the desired reaction time (4 h, unless stated otherwise) the reactor was rapidly cooled down in a water bath and the pressure was released. The liquid product mixture was filtered to separate the catalyst powder and analyzed by GC and HPLC. During selected experiments, additional samples were taken via the dip tube.

Liquid samples were analyzed by gas chromatography (GC) using a Shimadzu GC‐2010 with a CP‐Sil 8 CB column (Agilent) and a flame ionization detector. GC analysis was used to detect EG, acetic acid, ethanol and methanol. In addition, high‐performance liquid chromatography (HPLC) analysis of the liquid samples was conducted to detect GA and acetic acid. A Shimadzu Prominence HPLC (LC‐20 A) with a photodiode array detector (SPD‐M50A, set at 210 nm) was used in combination with a Nucleodur PolarTech column (Macherey‐Nagel). 5 mmol L^−1^ aqueous H_2_SO_4_ was used as mobile phase. For both GC and HPLC analysis external calibrations allowed quantification of all mentioned compounds. Based on this, GA conversion as well as yield and selectivity of the products were determined as follows in Equations (1), (2) and 3:

(1) $$\mathrm{Conversion}\;\mathrm{of}\;\mathrm{GA}\;X_{GA,\;t}=1-\;\frac{n_{GA,\;t}\;}{n_{GA,\;initial}}$$

(2) $$\mathrm{Yield}\;\mathrm{of}\;\mathrm{product}\;P\;Y_{P,\;t}=\;\frac{n_{P,\;t}\;}{n_{GA,\;initial}}$$

(3) $$\mathrm{Selectivity}\;\mathrm{for}\;\mathrm{product}\;P\;S_{P,\;t}=\;\frac{Y_{P,\;t}\;}{X_{GA,\;t}}$$

Elemental analysis by ICP‐OES was used to determine the amount of leached Ru during selected catalytic experiments. For that purpose, undiluted reaction mixture was analyzed using emission at 240.272 nm for quantification.

**Model solutions with additives and algal glycolate solutions**. The reference point for all catalytic results in this study is the experiment with a model solution of 70 mmol L^−1^ GA (Alfa Aesar, 98 %) in distilled water. To ensure that all discussed additive effects are significant, this reference experiment was repeated five times. From this, mean values and standard deviations for GA conversion (84±3 %), EG yield (39±11 %) and selectivity (46±11 %) were calculated (more details in Table S2).

Model solutions with additives were prepared by adding the respective compound(s) to the aqueous 70 mmol L^−1^ GA solution. An overview of all experiments and the additive concentrations used is listed in Table S3, entries 2‐31. The pH value of the GA solution was varied in the range of 1.4‐8 by adding acids (HCl, HNO_3_, H_2_SO_4_; entries 2–7) and bases (KOH, NH_3_; entries 8–13). Inorganic salts were added one at a time (NaCl, Na_2_SO_4_, NaNO_3_, NH_4_Cl, K_2_HPO_4_; entries 14–22). Nutrient salts (total concentration <1 mmol L^−1^) were added as a combined solution (entry 23, detailed in Table S4). Furthermore, two pH buffers were investigated (entries 24‐25): MES (2‐(*N*‐morpholino)ethanesulfonic acid) and TRIS (tris(hydroxymethyl)aminomethane). Other compounds studied were the complexing agent ethylenediaminetetraacetic acid (EDTA, entries 26–27) and the metabolic inhibitor EZA (entries 28–30). Concentrations of the model compounds (excluding the pH variation) were first based on the respective (estimated) concentrations in the real algal medium and in certain cases varied for further investigation.

Two AGAS from different photobiocatalytic experiments (see above) were studied (Table S3, entries 32–33, detailed composition in Table S4): AGAS 1 is derived from a photobiocatalytic experiment with metabolic inhibitor EZA and TRIS buffer and AGAS 2 from a second, novel approach without EZA and using MES buffer. To enable successful conversion, the pH value was adjusted from neutral to ca. 2.5 by addition of H_2_SO_4_, based on the findings for the pH variation with model solutions. For better comparison with the previously described model solutions, additional GA was added to increase its concentration from ca. 20 mmol L^−1^ to >70 mmol L^−1^. In this case, KOH was added to neutralize the added GA before adjusting the pH to 2.5 with additional H_2_SO_4_.

## Conflict of interest

The authors declare no conflict of interest.

1

## Supporting information

As a service to our authors and readers, this journal provides supporting information supplied by the authors. Such materials are peer reviewed and may be re‐organized for online delivery, but are not copy‐edited or typeset. Technical support issues arising from supporting information (other than missing files) should be addressed to the authors.

Supporting Information

## Data Availability

The data that support the findings of this study are available in the supplementary material of this article.
