# Supplementary material for: Ru/C‐Catalyzed Hydrogenation of Aqueous Glycolic Acid from Microalgae – Influence of pH and Biologically Relevant Additives
Source: ChemistryOpen. 2022 Jul 13;11(7):e202200050. doi: 10.1002/open.202200050 (PMC9278103; doi:10.1002/open.202200050)
Supplement: Supplementary file 1 — Supporting Information [file OPEN-11-e202200050-s001.pdf]

ChemistryOpen

Supporting Information

Ru/C-Catalyzed Hydrogenation of Aqueous Glycolic Acid from Microalgae – Influence of pH and Biologically Relevant Additives

Florian M. Harth, Joran Celis, Anja Taubert, Sonja Rössler, Heiko Wagner, Michael Goepel, Christian Wilhelm, and Roger Gläser*

Supplementary Material

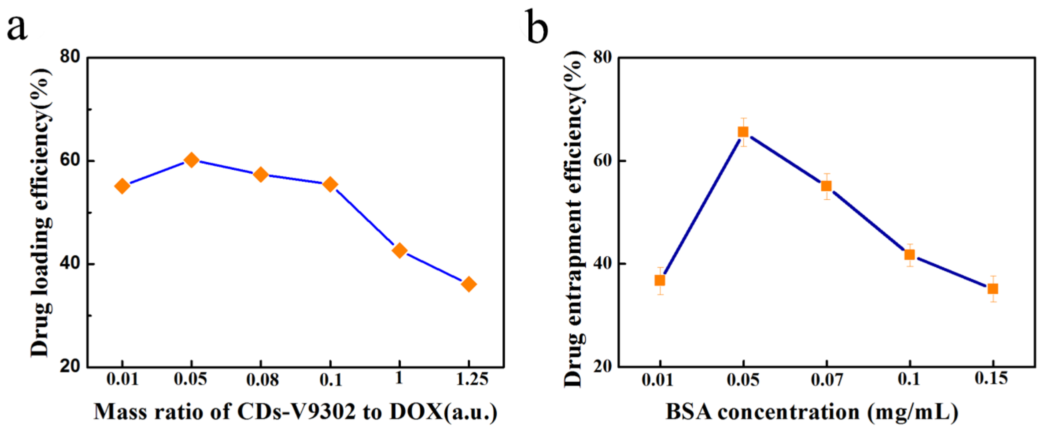

Fig. S1 (a) Drug loading rates of CDS-V9302 and DOX; (b) The encapsulation rate of BSA@CDs-V9302/DOX

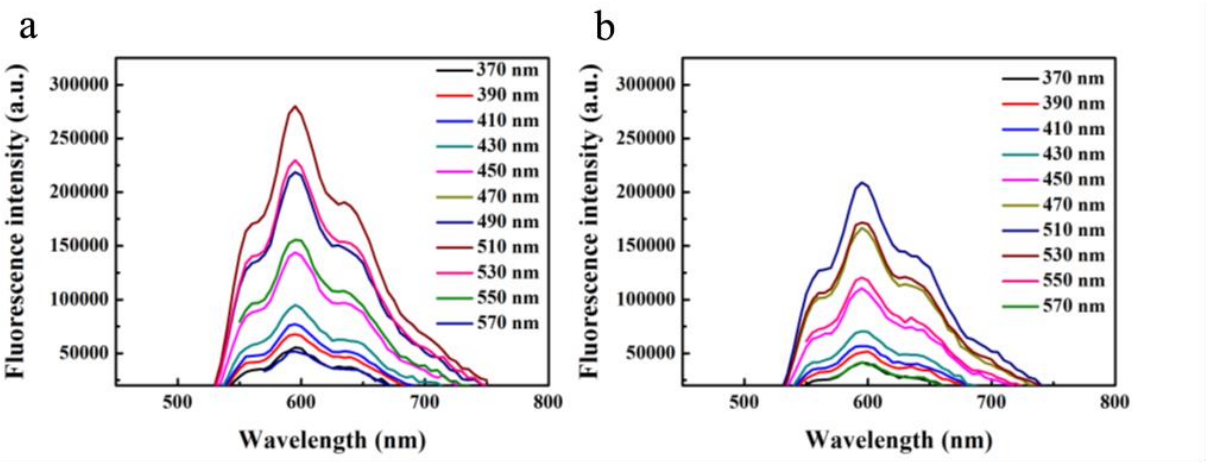

Fig. S2 Fluorescence spectra of (a) CDs-V9302/DOX and (b) BSA@CDs-V9302/DOX at different excitation wavelengths.

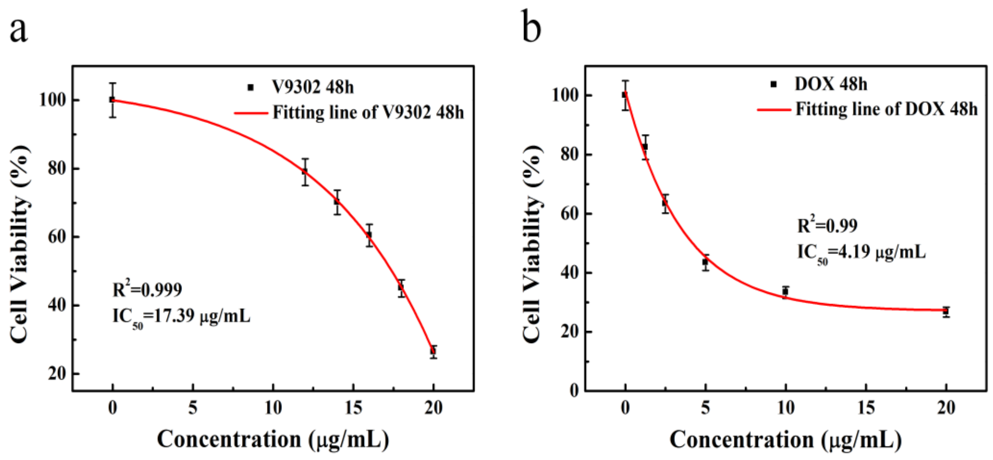

Fig. S3 Toxicity fitting effect of V9302 (a) or DOX (b) co-incubated with MGC-803 cells for 48 h.
